# 73. Identification of Novel Factors Associated with Inappropriate Treatment of Asymptomatic Bacteriuria Treatment in Acute and Long-term Care

**DOI:** 10.1093/ofid/ofab466.275

**Published:** 2021-12-04

**Authors:** Marissa Valentine-King, John Van, Casey E Hines-Munson, Laura Dillon, Christopher J Graber, Payal K Patel, Dimitri M Drekonja, Paola Lichtenberger, Bhavarth Shukla, Jennifer Kramer, David J Ramsey, Barbara Trautner, Larissa Grigoryan

**Affiliations:** 1 Baylor College of Medicine, Houston, Texas; 2 Michael E. DeBakey VA Medical Center, Houston, Texas; 3 VA Greater Los Angeles Healthcare System/UCLA, Los Angeles, California; 4 University of Michigan and VA Ann Arbor Healthcare System, Ann Arbor, MI; 5 Minneapolis Veterans Affair Health Care System, Minneapolis, MN; 6 University of Miami Miller School of Medicine and the Miami VA Healthcare System and University of Miami, Miami, FL; 7 University of Miami, Miami, Florida; 8 Michael E DeBakey VA Medical Center, Houston, Texas

## Abstract

**Background:**

Inappropriate treatment of asymptomatic bacteriuria (ASB) is a major driver of antibiotic overuse. Demographic and laboratory factors associated with inappropriate antibiotic treatment include older age, pyuria, leukocytosis and dementia. To gain a deeper understanding of inappropriate ASB treatment, we performed an in-depth review of provider documentation capturing a broader range of misleading factors associated with ASB treatment.

**Methods:**

We reviewed a random sample of 10 positive urine cultures per month per facility from acute or long-term care wards at eight Veteran’s Administration (VA) facilities from 2017-2019 (n=960). Trained chart reviewers classified cultures as UTI or ASB and as treated or untreated. Charts were searched specifically for mention of 8 categories of potentially misleading symptoms that often lead to overtreatment of ASB (e.g. “prior history of UTI”) (**Figure** legend). We also created a ‘suspected systemic inflammatory response syndrome (SIRS)’ category that included any mention of leukocytosis, tachycardia, tachypnea, subjective or low-grade fever, or hypothermia. Generalized estimating equations logistic regression was used for analysis.

**Results:**

Our study included 575 cultures from patients that were primarily white (71%) males (94%) from acute medicine units (75.7%) with a mean age of 76. Twenty-eight percent (n=159) of ASB cases received antibiotics. In addition to the usual known predictors, multiple new misleading symptoms were found to be associated with ASB treatment (**Table**). Novel, independent predictors of ASB treatment included behavioral issues, such as falls or fatigue (odds ratio (OR): 1.8; 95% CI: 1.05-3.07), urine characteristics, such as cloudy or odorous urine (OR: 1.41; 95% CI: 1.13-1.75), voiding issues (OR: 1.86; 95% CI: 1.43-2.41), and a single, free text mention of a SIRS criteria (OR: 1.63; 95% CI: 1.16-2.3).

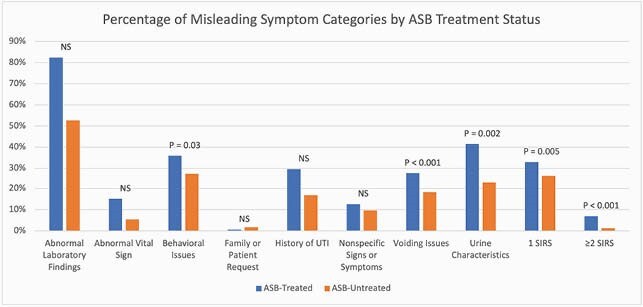

P-values extracted from multivariate regression model (ASB-asymptomatic bacteriuria; NS-not significant; SIRS- systemic inflammatory response syndrome). The following signs or symptoms compose each category: abnormal laboratory findings: acute kidney injury, abnormal creatinine, leukocytosis, pyuria/positive urinalysis, hyperglycemia; abnormal vital sign: bradycardia, tachycardia, atrial fibrillation, hypotension, hypertension, hypoxia, tachypnea, subjective fever or low-grade fever, syncope; behavior issues: falls, confusion lethargy, fatigue, weakness; nonspecific signs or symptoms: nonspecific gastrointestinal, genitourinary, neurological symptoms; voiding issues: decreased urine output, urinary retention, urinary incontinence; urine characteristics: change in color, foul smell, cloudy urine, sediment; SIRS: ordinal variable characterizing if 1 or ≥ 2 of the following were documented by the provider: leukocytosis, tachycardia, tachypnea, subjective or low-grade fever, hypothermia.

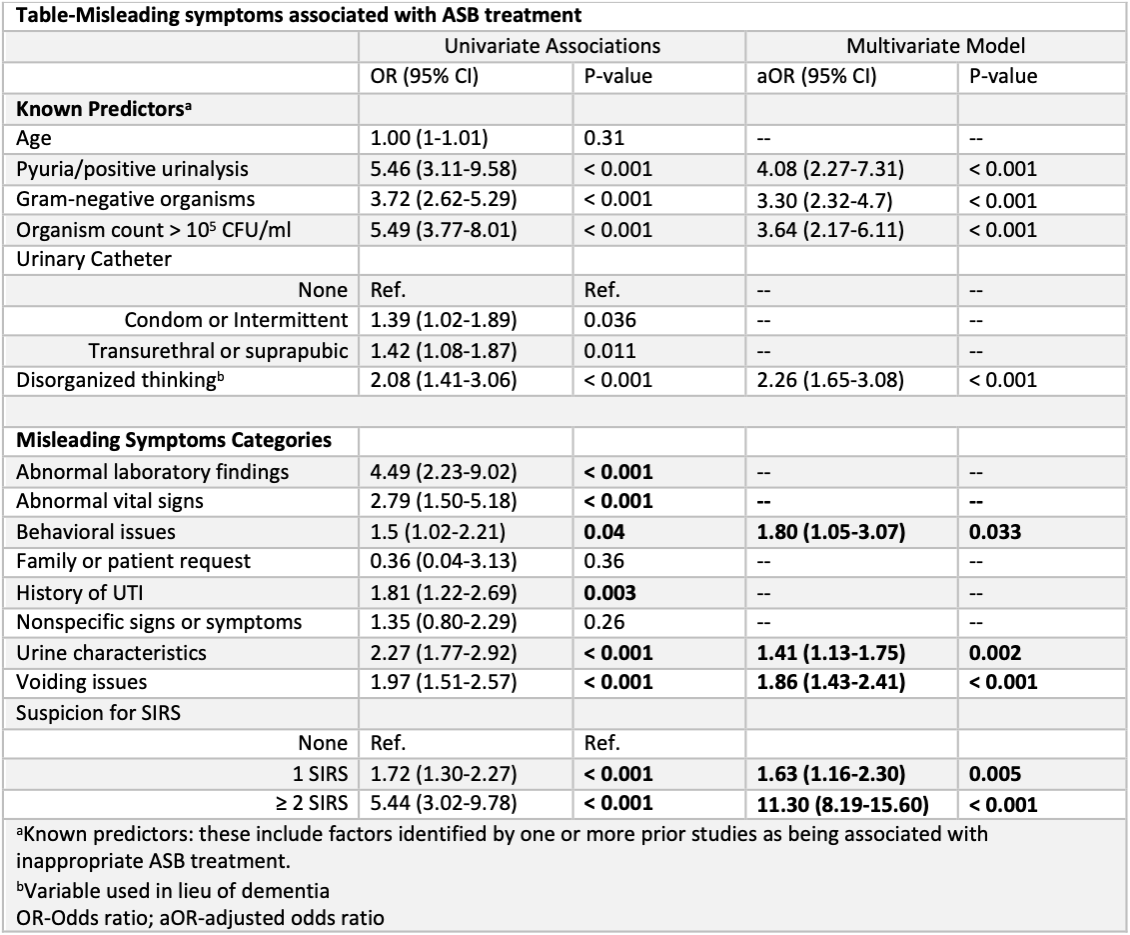

**Conclusion:**

Our in-depth chart review, with attention to misleading symptoms and any documentation of the provider thought process, highlights new factors associated with inappropriate ASB treatment. Patients with even a single SIRS criteria are at risk for unnecessary treatment of ASB; this finding can help design antibiotic stewardship interventions.

**Disclosures:**

**Barbara Trautner, MD, PhD**, **Genentech** (Consultant, Scientific Research Study Investigator)

